# Hereditary Angioedema: The Economics of Treatment of an Orphan Disease

**DOI:** 10.3389/fmed.2018.00022

**Published:** 2018-02-16

**Authors:** William Raymond Lumry

**Affiliations:** ^1^Allergy Division, Internal Medicine, University of Texas Southwestern Medical Center, Dallas, TX, United States

**Keywords:** health economics, orphan disease, hereditary angioedema, burden, treatment

## Abstract

This review will discuss the cost burden of hereditary angioedema on patients, healthcare systems, and society. The impact of availability of and access to novel and specific therapies on morbidity, mortality, and the overall burden of disease will be explored along with potential changes in treatment paradigms to improve effectiveness and reduce cost of treatment. The prevalence of orphan diseases, legislative incentives to encourage development of orphan disease therapies and the impact of orphan disease treatment on healthcare payment systems will be discussed.

Hereditary angioedema (HAE) is a rare autosomal-dominant genetic disease (1, 2). It occurs in approximately 1:30,000–80,000 individuals and affects less than 8,000 individuals in the United States (USA), 15,000 in Europe, and 200,000 worldwide (3, 4) Disease-specific therapy for HAE, although available in other countries since 1979 (5), only recently became available in the USA. With the Food and Drug Administration (FDA) approval of a human nano-filtered human plasma-derived C1 inhibitor administered intravenously for routine prevention of HAE attacks on October 10, 2008, a new era in therapy for HAE began (6). Since 2008, four therapies to treat HAE attacks have received FDA approval, including nano-filtered pasteurized human plasma-derived C1 inhibitor and ecallantide in 2009, icatibant in 2011, and recombinant C1 inhibitor in 2014 (7–11). These therapies are currently registered in many countries around the world. A concentrated human plasma-derived C1 inhibitor administered subcutaneously for routine prevention of HAE attacks was approved on June 22, 2017 by the FDA and other preventive therapies are currently in development (12).

This burst of drug development and approvals has greatly benefited individuals with HAE, their families, caregivers, physicians, and healthcare providers (13, 14). The burden of HAE has been reduced, quality of life improved and utilization of urgent care, emergency facilities, and hospitals decreased significantly (14, 15). Concerns raised by healthcare payers in the USA and healthcare systems in other countries about the financial impact of these newly approved therapies on healthcare payment systems have led to barriers to and limitation of access to these life changing and potentially life saving therapies (16–20).

This review will discuss the cost burden of HAE on patients, healthcare systems, and society. The impact of availability of and access to novel and specific therapies on morbidity, mortality, and the overall burden of disease will be explored along with potential changes in treatment paradigms to improve effectiveness and reduce cost of treatment. The prevalence of orphan diseases, legislative incentives to encourage development of orphan disease therapies, and the impact of orphan disease treatment on healthcare payment systems will be discussed.

## Impact of New Therapies for HAE

Patients with HAE have benefited from approval of novel, disease-specific drugs that treat and prevent swelling attacks. The benefits include improvement in health and quality of life (QoL), increased ability to work and pursue educational and career goals, reduced disability and reduction of costly urgent care visits, and hospitalizations and longer survival (13, 14, 15).

The economic cost of these therapies is high. In 2012, the annual cost of nano-filtered C1 inhibitor (Cinryze^®^, Shire, Lexington, MA, USA) indicated for routine prevention of HAE attacks, when used at approved dosage and interval, was $487,000 USD per patient, the most expensive drug for any orphan disease treated in the USA (21). In 2015, sales of Cinryze^®^ generated the second highest revenue per patient treated to a pharmaceutical company, $210,000 USD, of all orphan drugs (22). Current USA average wholesale prices (AWP) for treatments of swelling attacks range from $5,000 to more than $12,000 per attack treated. The cost of these therapies is generally less outside of the USA with some national health systems able to acquire them at a significant discount to USA prices.

Although not commonly seen in the orphan disease drug market the increasing number of approved therapies for HAE may result in competition between pharmaceutical companies decreasing the cost of these therapies. The first example of this occurred recently with the pricing of Haegarda^®^, a subcutaneous C1 inhibitor concentrate (C1INH) for routine prevention of HAE attacks approved in June, 2017. Despite approximately four times the amount of C1 inhibitor being required to treat a patient with Haegarda^®^ compared to Cinryze^®^, the AWP per treatment with Haegarda^®^ is 15% less. As other drugs are developed and approved for this indication hopefully competitive pricing will continue to push costs down.

In addition to the expense of specific HAE therapies, other costs of caring for an individual with HAE are high. These costs include the direct cost of providing medical care to these patients and the indirect costs of the disease on patients, their families, and on society. Patients with HAE consume more health care and are more costly to the health care system. In a United Kingdom study, the cost of care for an HAE patient was 160% more for primary care and 447% more for secondary care than a patient without HAE even if the costs of specialist care and medications were excluded (23). During swelling attacks, patients with HAE and their caregivers miss a significant amount of time from work and school, and when “well” are often less productive at work. Many report being unable to achieve their educational and career goals, or even maintain employment as a result of their disease (24, 25).

## Cost of HAE Care Before Specific Therapies were Avaliable in the USA

As disease-specific therapies for HAE were not available in the USA before 2008, there is an opportunity to review costs and impact of the disease before current therapies became available. An internet survey completed by members of the USA HAE Association (HAEA) in 2007 found patients experienced an average of 26.9 swelling attacks per year lasting 61.3 h each. Over 80% were considered moderate to severe in intensity (25). Total annual cost of having and treating HAE in the USA was also assessed in this survey (26). The average annual cost per patient was $44,597. Cost varied depending on the severity of the disease ranging from $11,587 for mildly affected patients to $28,764 and $104,857, respectively, for those moderately or severely affected. The total mean annual expenditure per HAE patient of $44,597 included direct medical costs of $29,177 comprised of $17,381 for hospital admissions, $2,827 for emergency department (ED) visits, $3777 for outpatient care, and $5,194 for medications and indirect costs of $15,420, including $5,157 for reduced productivity, $6,417 for reduced income, $3,402 for missed work, and $4,444 for travel and childcare (26). Accounting for inflation, the average annual cost per patient in 2017 would be more than $65,000.

Emergency department usage and hospitalization before 2008 was high as only supportive treatment, including intravenous fluids, anti-emetics, analgesics, and airway support was available. In 2007, there were 2,705 ED visits in the USA with HAE as the primary diagnosis with 40.9% resulting in hospitalization. At a mean cost of $1,479 per ED visit $3,727,080 was spent for urgent care (27). Between 2004 and 2007, there were 10,125 hospitalizations where HAE was the primary or secondary diagnosis. The average length of stay was 5.1 days and average cost per hospitalization was $8383 resulting in $21,220,000 annual expenditure for hospital services (28).

## Burden of HAE

In addition to the direct cost of medical care, indirect costs must be considered. Missed time from work or school, decreased productivity at work and loss of opportunity are significant costs to the patient and society. Patients report a decrease of productivity on the job due to HAE (26). A national audit in the United Kingdom of patients with HAE and acquired angioedema revealed patients lost on average 9 days of work per year ranging from 0 to 43 days (29). A European Union (EU) study of the socioeconomic burden of HAE revealed both patients and caregivers were affected with each losing an average of 20 days from work or school per year (24).

Loss of educational and career opportunity is commonly reported. In the HAEA survey conducted in 2007, 57% of HAE patients reported having career advancement hindered, 69% felt that they could not consider certain types of jobs because of their disease, 100% felt that educational advancement had been hindered, 55% had to limit their educational choices, and 48% had not achieved the level of education that they desired (25). Even after disease-specific therapies became available, decreased opportunity remains a problem. In an EU socioeconomic burden of disease report published in 2014 after current therapies became available, 42% patients reported their educational advancement was hindered, 40% were prevented from applying for certain jobs and 36% felt that their career advancement was diminished, 9% switched positions within their company, and 10% left their position permanently because of this disabling disease (24). HAE patients also suffer from anxiety and depression at much higher rates than the normal population. Results from two independent studies suggest that between 14 and 43% of patients of HAE experience clinically significant depression further adding to their cost of treatment and disability (25, 30).

A fatal HAE attack with loss of an individual both from the work force and from society is the ultimate burden of this disease and results in significant hardship both socially and economically to the patient’s family and society. Unfortunately, patients with HAE continue to succumb to this treatable disease. In a review of all USA death certificates from 1999 to 2010, HAE was considered a contributing factor or the underlying cause of death in 600 people with 270 of these deaths directed attributed to HAE (31). In a German study which included 728 patients from 182 families with HAE, 214 deaths were recorded. The mean age of death from an HAE attack was 40.6 years (32).

## Innovative Treatment Paradigms

Although disease-specific treatments for HAE are costly, appropriate and timely treatment decreases ED visits, hospitalizations, lost time from school and work, and prevents death lowering the overall cost of the disease to the healthcare system and to society. ED visits and hospitalization for supportive treatment were the norm before availability of home and self-administration of treatment (25). Self-administration for treatment and prevention of attacks is safe, effective and encouraged. Patients can accurately recognize and safely self-treat HAE attacks leading to earlier treatment, earlier resolution of symptoms, decreased ED and hospital visits, and improved quality of life and cost savings (33–39).

Innovative treatment paradigms may lower the cost and the burden of disease further. Italian HAE patients who self-administered C1INH decreased their mean annual number of hospitalizations from 16.8 to 2.1, time to administration of treatment from 3.2 to 1.9 h, time to beginning of symptom improvement from 84 to 54 min, time to complete symptom resolution from 12.8 to 10.8 h and number of missed days of work or school from 23.3 to 7.1 compared to hospital or ED administered therapy. The total cost of therapy, including both direct and indirect cost, was approximately 30,010 Euros ($36,000 USD) per patient when the therapy was administered in a hospital or clinic setting and 26,621 Euros ($32,000 USD) when treatment was administered at home. This represented savings of 11% or 57,619 Euros ($69,000 USD) for the 17 patients reported in the study (40).

Spanish investigators estimated patients who self-treated their HAE attacks with icatibant compared to health care setting administration would save an average of 121 Euros ($145) per attack in direct and indirect costs, a 9.2% decrease in costs. Reduction in direct costs accounted for 74% of the savings. This would achieve an annual health system savings of 551,371 Euros ($661,600) (41). An USA study reported a $650,000 savings when 249 HAE attacks over 5 months were treated with ecallantide given at home by an infusion nurse compared to the cost of treating these attacks in the ED or hospital (42). In the United Kingdom, home administration of icatibant compared to hospital administration of C1INH saved $861–$1167/attack (43).

In Denmark, 80 HAE patients were followed prospectively for 10 years. By 2012, 49% were self-administering C1NH or icatibant with 84% reduction in ED visits in this group. In the self-treated patients, there was no need for tracheotomy, no deaths reported and an improved QoL in all physical and psychological domains. Despite a 300% increase in use of newer “high cost” treatments, the cost to treat an average of 36 attacks per patient per year was a manageable at $16,766 (35).

Unfortunately in many countries, implementation of a home or self-administration policy is not possible. In Japan, Greece and most of Eastern Europe acute therapies are only available at the hospital or in specialty clinics, if available at all. In Brazil and Mexico, home therapy is available but is not reimbursed.

Specific treatments for HAE attacks are available in many countries as shown in Table 1. Treatments to prevent attacks are less widely available. What is not obvious from this table is although these treatments may be registered and approved for marketing in a particular country, they may not be accessible to the patients that need them. Barriers to access include requirement for health care system or judicial approval, availability only in specialized treatment centers or hospitals, limits on reimbursement, and limits on number of treatments allowed or resupply of medication to providers or patients.

**Table 1:** HAE Treatment Registration around the world – December, 2017.

Drug	Registration	Indication				Age/groups		Route
		Acute treatment	Prophylaxis		Home Therapy	Children, <12 years old	Adolescence, 12–18 years old	
			STP	LTP				
pdC1-INH (Berinert^®^)	Europe	✓	✓	–	✓	✓	✓	IV
	USA	✓	–	–	✓	✓	✓	IV
	Latin America (Brazil, Argentina, Mexico, Colombia, Chile, Puerto Rico)	✓	✓	–	✓	✓	✓	IV
	Australia/Cana da	✓	✓	–	✓	–	✓	IV
	Israel	✓	✓	–	✓	✓	✓	IV
	Japan	✓	✓	–	✓	–	✓	IV
	South Korea	✓	✓	–	✓	✓	✓	IV

rhC1-INH (Ruconest^®^)	Europe	✓	–	–	–	–	–	IV
	USA	✓	–	–	✓	–	✓	IV

pdC1-INH (Cinryze^®^)	Europe	✓	✓	✓	✓	–	✓	IV
	USA	–	–	✓	✓	–	✓	IV
	Australia/Canada/Israel	✓	✓	✓	✓	–	–	IV

pdC1-INH SC (Haegarda^®^)	USA			✓	✓		✓	SC

Icatibant (Firazyr^®^)	Europe	✓	–	–	✓	–	–	SC
	USA	✓	–	–	✓	–	–	SC
	Latin America (Brazil, Argentina, Mexico, Colombia)	✓	–	–	✓	–	–	SC
	Australia/Canada	✓	–	–	✓	–	–	SC
	Israel/Kuwait/South Africa	✓	–	–	✓	–	–	SC

Ecallantide (Kalbitor^®^)	Europe	–	–	–	–	–	–	SC
	USA	✓	–	–	✓*	–	✓	SC

Attenuated androgens	Europe	–	✓	✓	✓	–	✓	Oral
	USA	–	–	✓	✓	–	–	Oral
	Latin America (Brazil, Argentina, Mexico, Colombia)	✓	–	–	✓	–	–	Oral

Tranexamic Acid	Europe	–	✓	✓	✓	✓	✓	Oral
	USA	–	–	–	✓	–	–	Oral
	Latin America (Brazil, Argentina, Mexico, Colombia)	✓	–	–	✓	–	–	Oral

Epsilon amino caproic acid	Europe	–	–	–	✓	–	–	Oral
	USA	–	–	–	–	–	–	Oral
	Latin America (Brazil, Argentina, Mexico, Colombia)	–	–	–	–	–	–	Oral

*STP, short term prophylaxis; LTP, long term prophylaxis*.

*Not for self-administration.

An example of this problem is found in Argentina. Members of the Argentine HAE Patient Association completed questionnaires about the availability and their access to HAE treatments in 2009, 2013, and 2016. Despite C1INH being registered and approved for treatment of HAE attacks before 2009, C1INH was available to only 26% of those responding to the 2009 survey. This increased to 55% by 2016. Despite being approved only 10% had access to icatibant in 2016. Reimbursement for these medications in 2016 was also a challenge with only 64% reimbursed at 100% with 19% having no reimbursement available at all. The majority of the patients received treatment from health personnel or at the hospital and over 50% reported not receiving treatment until their attack was severely painful. Reordering and resupply of medication was difficult for 66% of patients with only 20 per reporting a fast replacement and 53% being able to obtain replacement within 10 days (44).

Despite the advances in the treatment for HAE with availability of new effective on demand and attack preventing treatments, these medications are not approved in many countries around the world. Even if approved, they are often not accessible to the patients who need them. Despite improvement of treatment outcomes and cost savings, when the treatment is self-administered or given in the home setting, in many countries treatment of attacks is only available in hospital and clinic settings.

## Orphan Drug Development Policies and Reimbursement Issues

Individuals with HAE and other rare and burdensome diseases have benefited greatly from the development of therapies made economically feasible by the orphan drug policies. Orphan drug policies have been established in the USA, the EU and Japan to encourage the development of safe and effective therapies for rare diseases. The USA Orphan Drug Act was enacted in 1983 (45). Under this act, a drug is given orphan designation if the disease it treats affects less than 200,000 (approximately 1:1,650) individuals or if there is no reasonable expectation of profitability for the drug. Incentives provided include tax credits for research costs, grants to aid in clinical research, and a 7-year marketing exclusivity for approved orphan drugs.

In 1999, the EU enacted its orphan drug policy that defines an orphan disease as one with 5 or less patients per 100,000 (1:20,000) individuals. Research incentives are available within the EU and its member States and fees are waived for approval of the marketing application and a 10-year marketing exclusivity is provided (46).

These policies have been successful in encouraging companies to develop therapies for a wide variety of rare conditions. In January, 1983 there were 38 drugs approved in the USA for orphan disorders (22). On July 1, 2017, 635 approved drugs were listed in the Orphan Disease Therapeutic Registry (47). and 98 designated orphan drugs were approved for marketing in the EU (48).

There are 25–30 million individuals, 8–10% of the population of the USA, affected by one of the 7,000 diseases designated as orphan, who may benefit from provisions of the Orphan Drug Act (16, 49). Although treatments for these rare diseases have provided great benefits to affected individuals and their families, the high cost of these therapies has led to the perception by payers and some in society that treatment of orphan diseases places an inordinate burden on healthcare payment systems (16, 17). This concern about high cost of these treatments has resulted in barriers to limit access to them, including formulary approval, high coinsurance and copayment rates, prior authorization and multiple reauthorizations, step therapy, and limits on supply and resupply of medication (16, 17, 20).

Drug research and development are time-consuming and expensive activities. The average cost of developing and winning market approval for a new prescription drug was recently estimated to be $2.6 billion with $1.4 billion of this amount spent on research and development (50, 51). Orphan drugs are no exception. With many fewer patients to treat, the cost per patient treated must be higher in order to recover research and development expenditures. For the top 100 drugs in 2016, the annual median drug cost per patient in the USA for treatment of non-orphan conditions was $27,756 compared to $140,443 per patient treated with an orphan disease (49).

Although the cost per patient to treat an orphan disease is often high, the perception that cost of treatment of rare diseases as a whole has an inordinate impact on total pharmaceutical expenditures and health care costs is inaccurate. Analyses in the USA and EU have shown this impact is minimal and in line with the 8–10% prevalence of these diseases in the population. In 2014, total expenditures for pharmaceuticals in the USA accounted for only 9.8% of total healthcare expenditures of $3.0 trillion. Expenditures on orphan drugs for orphan indications was approximately $33.5 billion representing less than 10% of pharmaceutical expenditures and only 1% of the total healthcare expenditures (22, 52).

Orphan drug expenditures have increased over the last decade due to the increasing price of these therapies and the number of them registered and approved. In 2007, $13.3 billion was spent in the USA accounting for 4.3% of $311 billon total expenditures for pharmaceuticals that year. By 2013, this had increased to $25.8 billion, 7.7% of $337 billion pharmaceutical spend. IMS Health Market Prognosis has forecasted USA total drug expenditures of $465.0 billion in 2018 with orphan drugs accounting for $44.10 billion, 9.5% of this amount, representing an increase of 0.7% over four years (52, 53).

## Summary

Orphan drug policies are in place to encourage development of safe and effective therapies for orphan diseases. With a limited number of patients to treat, the cost per patient of orphan drugs to recover research and development costs is high. Orphan diseases affect approximately 8–10% of the population. Overall expenditures for drugs to treat orphan diseases remain proportional to the incidence of these diseases in the population. Expenditures for orphan drugs are currently less than 10% of pharmaceutical expenditures and 1% of the total healthcare costs in the USA. Despite these facts, payers in the USA and healthcare authorities around the world perceive treatment of orphan diseases puts an inordinate burden on their payment systems and have put barriers in place to limit use of new disease specific therapies.

Orphan drugs benefit many people with previously underserved orphan diseases. These drugs offer significant value to patients and society in terms of improvements in health, reduced disability, increased productivity, including the ability to continue working, reduced healthcare utilization, and improved quality of life and survival. Patients with HAE are not an exception and have benefited greatly. Availability of new and novel therapies to treat and prevent swelling attacks has dramatically decreased the burden of this disease. As the number of approved therapies for HAE increases the cost of therapy may decrease in response to competition. The first example of this occurred with the pricing of Haegarda^®^, a subcutaneous C1INH for routine prevention of HAE attacks approved in June, 2017. Prudent therapeutic choices, utilization of new treatment paradigms, and improvement in availability and accessibility to these new therapies will continue to improve the lives of patients with HAE and other orphan diseases. Coverage by payers and healthcare systems of these life altering and potentially life saving therapies should continue with barriers to access being appropriately addressed.

The conversation about the high cost for benefit obtained of orphan drugs with payers, society, and the patients needs to change to recognition of the benefits of timely and appropriate care. We need to solve the access problem by working with health systems, patients, and their advocates on the most cost-effective and efficient ways to deliver this care. We need to continue to disseminate knowledge regarding benefits to patients and to society of effective and safe treatments for HAE and all rare diseases.

## Author Contributions

WL researched and wrote the manuscript.

## Conflict of Interest Statement

WL is a consultant for biocryst, pharming, CSL Behring, and shire. He speaks on behalf of CSL Behring, pharming and shire and has received research grants from biocryst, pharming, dyax, lev, jerini, viropharma, CSL Behring, and shire. He is a member of the medical advisory board of the us hereditary angioedema association.
